# A framework for fused deposition modelling process evaluation using multivariate process capability analysis

**DOI:** 10.1371/journal.pone.0308380

**Published:** 2024-12-05

**Authors:** Moath Alatefi, Abdulrahman M. Al-Ahmari, Abdullah Yahia AlFaify, Mustafa Saleh

**Affiliations:** Industrial Engineering Department, College of Engineering, King Saud University, Riyadh, Saudi Arabia; Egaz Moniz School of Health and Science, PORTUGAL

## Abstract

The rapid advancement of additive manufacturing (AM) requires researchers to keep up with these advancements by continually improving the AM processes. Improving manufacturing processes involves evaluating the process outputs and their conformity to the required specifications. Process capability indices, calculated using critical quality characteristics (QCs), have long been used in the evaluation process due to their proven effectiveness. AM processes typically involve multi-correlated critical QCs, indicating the need to develop a multivariate process capability index (MPCI) rather than a univariate capability index, which may lead to misleading results. In this regard, this study proposes a general methodological framework for evaluating AM processes using MPCI. The proposed framework starts by identifying the AM process and product design. Fused Deposition Modeling (FDM) is chosen for this investigation. Then, the specification limits associated with critical QCs are established. To ensure that the MPCI assumptions are met, the critical QCs data are examined for normality, stability, and correlation. Additionally, the MPCI is estimated by simulating a large sample using the properties of the collected QCs data and determining the percent of nonconforming (PNC). Furthermore, the FDM process and its capable tolerance limits are then assessed using the proposed MPCI. Finally, the study presents a sensitivity analysis of the FDM process and suggestions for improvement based on the analysis of assignable causes of variation. The results revealed that the considered process mean is shifted for all QCs, and the most variation is associated with part diameter data. Moreover, the process data are not normally distributed, and the proposed transformation algorithm performs well in reducing data skewness. Also, the performance of the FDM process according to different designations of specification limits was estimated. The results showed that the FDM process is incapable of different designs except with very coarse specifications.

## 1. Introduction and literature review

Additive manufacturing (AM) has many advantages and increasing applications over traditional manufacturing methods. Therefore, its capability of producing products according to pre-determined specifications should be evaluated. The research in this study is based on the Fused Deposition Modeling (FDM) process due to its wide applications [1,2]. However, FDM also has some limitations, such as low resolution, rough surface finish, and poor dimensional accuracy [3,4]. In this regard, many studies have been published to monitor and evaluate the quality of AM processes [5]. Therefore, the final quality of 3D-printed objects can be evaluated using process capability analysis.

Process capability is the ability of a process to produce outputs that meet the specifications or requirements of the customer. In AM, process capability is important to ensure the quality of the products, as well as to reduce waste, cost, and time. AM process capability is affected by many factors, such as material properties and characteristics, machine parameters and settings, environmental conditions, and post-processing operations. In this regard, Razvan U and Ion [6] used statistical quality tools to estimate the process capability of the stereography process using two quality characteristics. However, they used a univariate capability index. Using a univariate capability index in the existing correlation has been proven to have misleading results [7]. Also, Bianca et al. [8], investigated the opportunities and challenges of applying statistical quality tools for AM processes. Moreover, statistical models are used to predict the shape of different products using a single model [9]. Scimone et al. [10] proposed a statistical modeling of deviations in AM process outputs. One recent trend is embedding sensors in the process and gathering a large sample of data during the additive manufacturing process [11]. Furthermore, Montgomery’s work on Statistical Quality Control provides a foundational understanding of process capability analysis, serving as a basis for its application in AM [12]. Several unique challenges in AM necessitate specialized approaches to process capability analysis. In this regard, Gao et al. (2015) highlighted the impact of layer thickness variation and build orientation on the process capability of AM [13]. In this regard, Lara R. & Irene F. [14] concentrated on the benchmark artifacts for assessing the geometrical performance of AM processes. However, research efforts have been directed towards developing methodologies for measuring and analyzing process capability in AM; these researches are limited and ignore using MPCI. Using these methods, additive manufacturing can achieve higher levels of process capability and quality. By achieving high levels of process capability and quality, additive manufacturing can satisfy customer needs and expectations and gain a competitive advantage in the market.

Ensuring the quality and consistency of AM-produced parts remains a significant challenge. Process capability analysis, a cornerstone of quality control in manufacturing, plays a pivotal role in assessing and improving the performance of AM processes. Montgomery’s work on Statistical Quality Control provides a foundational understanding of process capability analysis, serving as a basis for its application in AM [12]. Several unique challenges in AM necessitate specialized approaches to process capability analysis. Gao et al. (2015) highlighted the importance of understanding and mitigating factors such as layer thickness variation and build orientation, which can significantly impact the process capability of AM [13]. However, research efforts have been directed towards developing methodologies for measuring and analyzing process capability in AM. These researches are limited and ignore using MPCI. In this regard, Lara R. & Irene F. [14] concentrated on the benchmark artifacts for assessing the geometrical performance of AM processes and suggested a thorough analysis of the literature that is already in existence, carefully examining the design of such test pieces. The analyzed test components are divided into groups based on the aspects of the manufacturing process that they may assess.

Although there are many proposed MPCIs in the literature, no research has used them to evaluate any of the AM processes. An example of these proposed MPCIs, Wang and Chen [15] employed the main component analysis for the first time in their process capacity study. Principal components, or a set of uncorrelated linear functions, are created via PCA by transferring the associated QCs data. Using a variety of criteria, this approach reduces the dimension of the quality features. The specification limits (USL and LSL) are then subjected to the same transformation, changing the process’s overall coordinate axes [16]. Chen [17] introduced multivariate PCIs and established a comparison between the original PR SR. Chen did not include the PR’s place inside the SR in his research, despite the fact that he contrasted the PR with the SR. Das and Dwivedi [18] employed the g and h multivariate process capacity index for non-normal data. Their suggested PCI performs well when compared to other PCIs in the literature. Nevertheless, locating this PCI requires a lot of computational power. A multivariate PCI that may be used for both normal and non-normal QCS was proposed by Ciupke [19]. He recommends using on-sided models in the evaluation of multivariate non-normal processes in order to find the PR, which is then contrasted with SR.

Moreover, A study of dimensional characteristics was conducted to assess the FDM process’s suitability for component manufacture [20]. Other artifacts have been published in the literature using multiple materials and AM processes to evaluate and compare the capabilities of different AM processes [21]. Also, Loose and Nakagawa [22] designed an artifact comprised of extremely basic little features that may be tested for geometrical and dimensional accuracy as well as the capability of the manufacturing process to produce fine details. Furthermore, Xu et al. [23] established an artifact to test the capability of four different AM processes. These are stereolithography (SL), selective laser sintering (SLS), FDM, and laminated object manufacturing (LOM). The artifacts in the literature have many purposes according to the different characteristics, processes, and materials. The investigated quality characteristics are dimensional accuracy as in [24,25], repeatability [26,27], and minimum feature size [21,28]. Also, many artifacts were tested for an FDM process [29–31], whereas others were tested for many processes [32–34] in addition to the artifacts that were designed to test a specific type of materials such as ABS [35,36].

According to the above literature review, the existing studies that evaluate AM processes did not establish systematic approach for evaluating the capability of AM processes to produce parts within specification limits. However, many artifacts have been designed and produced for the evaluation process, and only short-term comparisons between nominal and measured QCs were made. That is by comparing all data points to the nominal dimension, without taking into consideration the variation of the process and its long-term effect. Long-term effect is reflected by the use of process capability indices, which are used in very limited studies in literature. Using the process capability index for multiple QCs required the researchers to investigate the existence of a correlation between QCs. Such that using a univariate capability index for correlated data reveals misleading results. To the best of the author’s knowledge, no study has investigated the correlation between QCs while estimating the process capability index for AM processes. Moreover, most of the real industry data are not normally distributed, so they need to be transformed using an appropriate transformation method. Therefore, this research presented a general framework for evaluating AM processes using a single multivariate process capability index.

The objective of this research paper is to comprehensively evaluate the performance of AM processes through multivariate process capability analysis. This entails a holistic assessment that considers multiple key process variables simultaneously, providing a more accurate representation of the manufacturing process’s true capability, in addition to the investigation of key variation factors that influence process capability in AM. The research in this article considered FDM process capability in producing specific cylindrical part within a given specification limits. By fixing FDM process parameters, the process output’s dimensional properties are evaluated against nominal dimensions. Therefore, the performance of FDM process is evaluated using process outputs.

The next section of this research presents the proposed methodology. The results are presented in Section three. Section four discusses the results of the study and the analysis of different alternatives for improvements. Finally, the research is concluded in section five.

## 2. Process and material

### 2.1 Process

In this research, the FDM process is evaluated because of its materials versatility, ease of usage, low price, and high precision. In FDM, a filament made of the polymer substance is melted in a heating chamber and introduced into the system. The tractor wheel-like apparatus that forces the filament into the chamber provides the extrusion pressure, which is what causes the pressure. Shortly after reaching the melting point, the temperature at which the material is manufactured causes solidification to begin.

**Fig 1:**
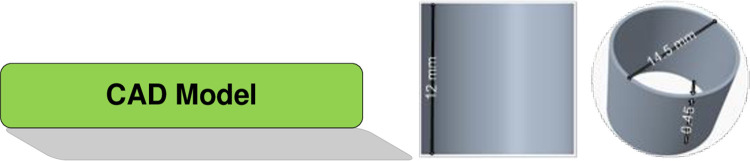
Additive manufacturing workflow.

For the purpose of evaluating the FDM process, a sample part has been designed with specific characteristics. The considered part has specific dimensions, as shown in Fig 1. Product design starts with 3D CAD modeling, as shown in Fig 1, followed by an STL file. In additive manufacturing, STL files are typically used to describe the surface of the planned object’s tessellation. The item is sliced into horizontal layers as the procedure proceeds. The end product is a machine-readable file that includes all the instructions needed to create the object using additive manufacturing.

The product was created using the STL format and CREO Parametric 8.0 software. To slice and create "Gcode" files, the STL files of the intended samples were exported from the Creo 8.0 program and loaded into PrusaSlicer 2.5.0. Table 1 is a list of the printing settings utilized. Table 1 is a list of the printing settings utilized. These settings were chosen based on the supplier’s recommendations for the used material (PLA) [37], the printer’s capabilities, and preliminary experiments. The extruder temperature of 215°C falls within the optimal range for printing PLA (210°C ±10), ensuring good flow during extrusion and adhesion between layers. A low extrusion temperature would increase the viscosity of the material, making it difficult to extrude and resulting in weak prints [38]. Conversely, an extrusion temperature that is too high would make PLA less viscous, leading to dimensional instability. The bed temperature was set to 60°C, within the recommended 40–60°C range, to ensure better adhesion between the printing bed and the first layer. The printing speed of 45 mm/s achieves an acceptable balance between speed and quality, and it is within the capabilities of most FDM printers. Similarly, the 0.2 mm layer thickness balances printing time and quality. Lower layer thickness would provide better resolution at the cost of increased printing time. The 0.2 mm layer thickness is also compatible with the 0.4 mm nozzle diameter, ensuring effective material deposition.

**Table 1:** FDM process printing parameters.

Parameter	Value
Extruder temperature, °C	215
Bed temperature, °C	60
Printing speed, mm/s	45
Layer height, mm	0.2
Infill, %	100

**Fig 2:**
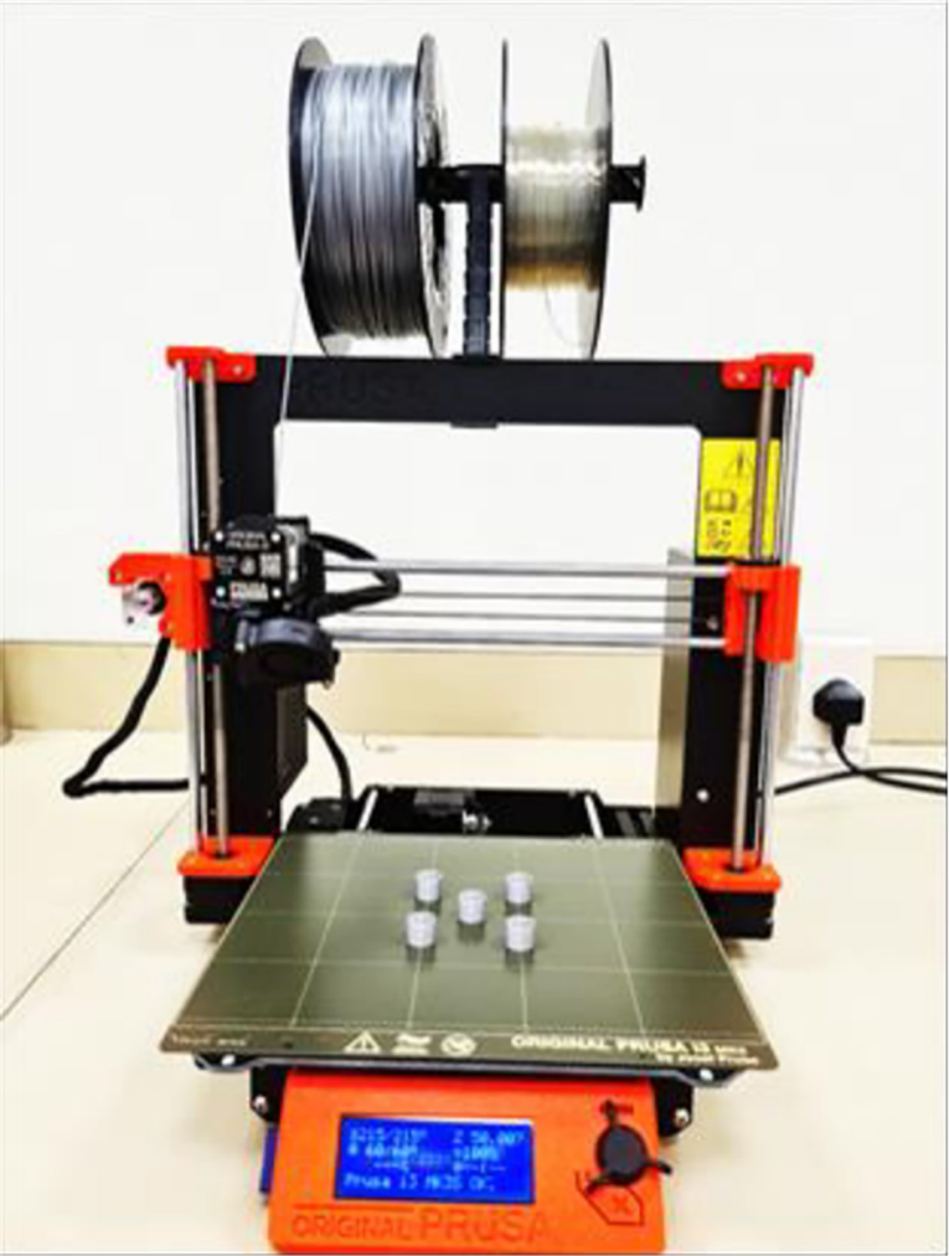
Utilized 3D printing machine.

The samples were produced using an open-access FDM printer dubbed an Original Prusa i3 MK3S+ printer with a 0.4 mm nozzle diameter (Fig 2).

### 2.2 Material

The material used to produce the required samples of the selected part is polylactic acid (PLA). A thermoplastic polyester known as PLA was first produced by the condensation of lactic acid and the evaporation of water. Alternately, lactide can be polymerized to create it using a ring-opening process. The affordability with which PLA can be produced from renewable resources has boosted its acceptance as a material. Despite not being considered a commercial polymer, PLA was the second-most popular bio-plastic globally in 2010. It has not been used widely due to a number of structural and operational difficulties [39]. It is the perfect material for this usage due to its low melting point, substantial strength, strong layer adhesion, moderate thermal expansion, and extreme heat resistance while annealed. Contrarily, when it has not yet been annealed, PLA has a low heat resistance among the main 3D printing polymers. The material used in this study is a 1.75 mm-diameter PLA filament (Prusament PLA Galaxy Silver filament), which was purchased from Prusa Polymers in the Czech Republic.

### 2.3 Additive manufacturing quality characteristics

Dimensional accuracy is critical in 3D printed parts because it determines how well the parts match the design specifications and how well they fit together with other components. If the parts are too large or too small, they may not function properly or cause problems in the assembly. Dimensional accuracy can be affected by many factors, such as the quality of the filament, the temperature of the extruder and the bed, the speed of the printer, and the calibration of the axes. The 3D-printed partsproduced for this study have many quality characteristics. However, the investigation is limited to the dimensional accuracy. Therefore, three-dimensional characteristics are determined in the design and are later measured in the printed specimens. These QCs are the height, diameter, and wall thickness of the samples. The nominal dimensions of height, diameter, and wall thickness are 12 mm, 14.5 mm, and 0.45 mm, respectively.

### 2.4 Additive manufacturing specification limits

Specification limits are the acceptable ranges of values for the dimensions of the parts produced by a specific process. Constructing specification limits requires identifying the relevant standards and regulations that apply to your AM process and product. For example, the ISO/ASTM standards on Qualification principles for AM processes and production sites. In this regard, the ISO 2768–1 standard [40] is used for dimensional tolerance, in addition to the appropriate statistical methods and tools for establishing specification limits based on the data. Tolerance intervals can be used to set upper and lower limits for your product characteristics and properties. As AM processes or 3D-printed parts change, the specification limits should be reviewed. These methods use mathematical formulas and data analysis tools to calculate the upper and lower limits for the dimensions of the parts based on the mean, standard deviation, and tolerance interval. For example, one can use the following formula to calculate the specification limits for a dimension L:

$$L=\bar{X}\pm kS$$

Where $\bar{X}$ is the mean value, k is a constant that depends on the confidence level and sample size, and *S* is the standard deviation. Upon this discussion of stating specification limits for AM processes, the research can use two different methods. The first method uses the published related standards to state the specification limits of the considered AM process based on material and nominal dimensions. Alternatively, the specification limits can be established based on the process outputs. In this regard, the natural confidence interval could be used. The natural confidence interval is calculated by adding ±3*σ* to the process mean.

## 3. Research methodology

**Fig 3:**
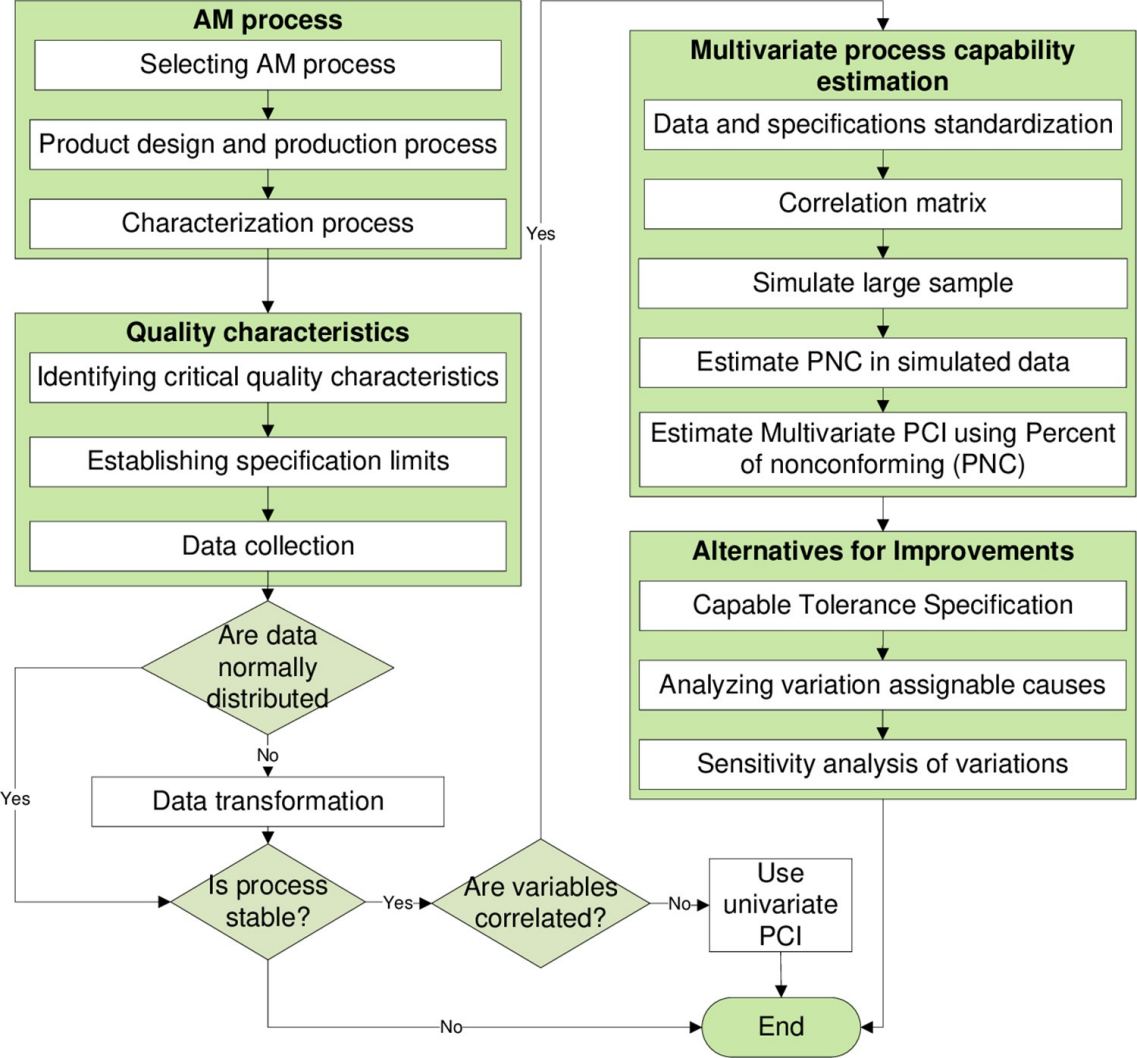
Research methodology.

This research proposes a general methodology that can be used to evaluate any AM process based on the 3D-printed part characteristics. Therefore, researchers and industry practitioners can use this framework regardless of the considered AM process. However, it is crucial to identify the process under investigation in order to come up with a systematic evaluation that leads to real findings. Consequently, the proposed methodology is explained and applied to the FDM process, as shown in Fig 3.

### 3.1 Data collection

**Fig 4:**
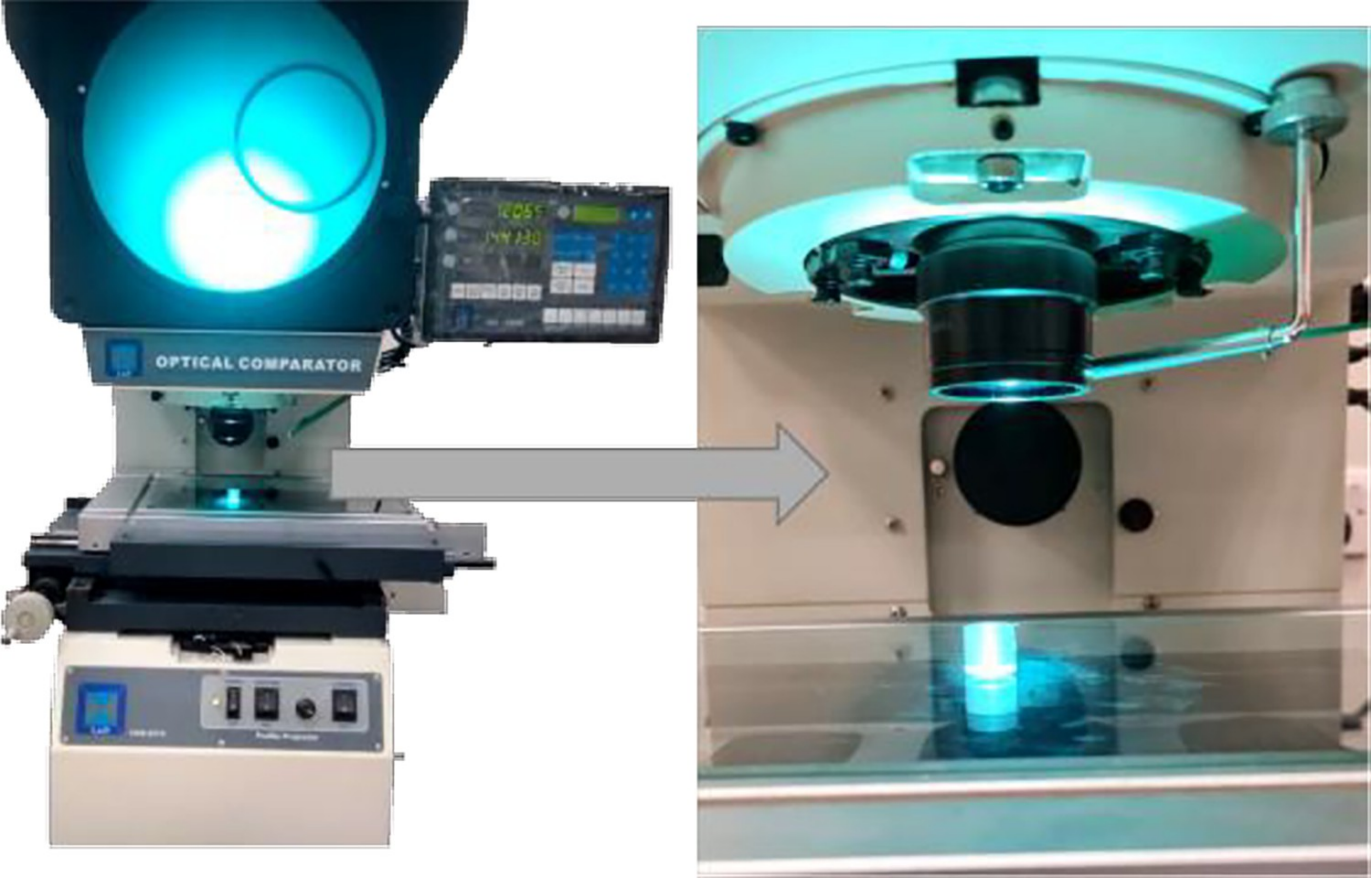
Profile projector.

The printing process has been operated in ten samples with 5-sample size each. Process parameters were fixed for each sample. Consequently, 50 specimens were 3D printed under the same conditions. Fixing process parameters is essential for process stability investigation. Th is, the process should be proven to be stable before conducting any process capability analysis. Therefore, the specimens were printed in different samples, so the control chats could be drawn. Upon completion of the printing process, the pre-determined QCs have been measured for all specimens. As mentioned above, these QCs are height, diameter, and wall thickness. The measurement process is done using a profile projector. This projector line is well-known for having a superior optical mechanism. High-quality picture processing and precise amplification are used. Under light, the contour scanning error is less than 0.08%. The coordinate indication deviation is less than (3+L/75) m, where L is the dimension of the objects to be measured in millimeters. The brand name of the measuring device used is L&D, and it is shown in Fig 4.

### 3.2 Normality, stability and correlation assumptions

To evaluate the performance of a process using process capability index, the process should have normal data and be proved to be statistically under control. Moreover, when the process has more than one correlated QC, multivariate process capability indexes are used instead of univariate capability index. The stability of the considered process has been presented in a previously published article for the same process, part, and data [41]. The mentioned paper is intended to investigate monitoring of the AM process with multivariate QCs. However, the considered research focuses on the second phase of process control, which is process evaluation using the process capability index. Moreover, the correlation between process QCs was statistically stated using the collected data, in addition to the functional correlation between QCs. Therefore, this part aims to evaluate the normality assumption of the considered process data and suggest a transformation method in case of finding non-normal data. This study uses the skewness property of the data to evaluate the normality assumption. That is normally distributed data has zero skewness. Any positive or negative value of skewness indicates a departure from normal distribution. Consequently, the process data skewness is calculated for each QC data and evaluated against an acceptable range of skewness. The skewed data, either to the right or the left, will be transformed to a normal distribution using the appropriate transformation method.

### 3.3 Multivariate process capability estimation

As stated earlier, most3D printed parts include more than one correlated QC. As this is the case, this study presents a multivariate capability index estimation algorithm. The algorithm initially standardizes the normal or transformed QCs data using the Eq 1 [42]:

$$Z_{i}=\frac{Y_{i}-{\hat{\mu }}_{Y_{i}}}{{\hat{\sigma }}_{Y_{i}}};\;i=1,2,\ldots ,p$$ (1)

Where *Y*_*i*_ the normal or transformed data, and p is is the number of the QCs, ${\hat{\mu }}_{Y_{i}}$ is the estimated mean of *QC*_*i*_ data, and ${\hat{\sigma }}_{Y_{i}}$ is the estimated standard deviation of *QC*_*i*_ data. Also, the specification limits are standardized using the mean and standard deviation of the process data Y_i_. To this point, all the QCs data follow a standard normal distribution. Therefore, the data forms a multivariate normal data with zeros mean vector ${\hat{\mu }}_{Z}=[0\;0\ldots ]$ and correlation matrix $\hat{\Sigma_{z}}$. These properties of the multivariate normal distribution are used to generate a large sample size from standard multivariate normal distribution in order to reach the steady state of the process. It is worth noting that the algorithm of estimating multivariate data has been developed using a Matlab package. Then, each generated standard multivariate normal vector is compared with the specification limits vector. Any generated vector out of the specification limits is recorded as nonconforming. Consequently, the total nonconforming vectors are divided by the sample size at the end of the generation process to estimate the percentage of nonconforming (PNC) as in Eq 2 [12]:

$$PNC=\frac{Number\;of\;nonconforming\;vectors}{Sample\;size}$$ (2)

Finally, PNC is used to estimate the potential multivariate process capability index using Eq 3 [42]:

$$C_{p}\frac{\varphi^{-1}(0.5+0.5(1-PNC))}{3}$$ (3)

Where *φ*^−1^ is the inverse cumulative density function of standard normal distribution (CDF). In the case of one-sided processes (processes with only upper or lower specification limit), the above equation is modified for the upper-sided process as in Eq 4 [42]:

$$C_{pu}=\frac{\varphi^{-1}(1-{PNC}_{u})}{3}$$ (4)

Where *PNC*_*U*_ is the percentage of nonconforming vectors above the upper specification limit. Moreover, the actual multivariate process capability could be calculated using Eq 5 [12]:

$$C_{pk}=Min(\frac{\varphi^{-1}(1-{PNC}_{U})}{3},\frac{\varphi^{-1}(1-{PNC}_{L})}{3})$$ (5)

Where *PNC*_*L*_ is the percentage of nonconforming vectors above the upper specification limit. The sample size of generated standard multivariate normal distribution data is 1,000,000, and the generation process is repeated 1000 times. In each replication, the multivariate process capability index is estimated, and then the average multivariate PCI is presented.

### 3.4 Process evaluation and sensitivity analysis

Established multivariate capability indices, *C*_*p*_ and *C*_*pk*_, are used to assess the capability of an AM process to produce products that meet predefined specifications. Acceptable values for these indices depend on the specific quality requirements and tolerances for the product in question. *C*_*p*_ Measures the potential capability of a process to produce products within the specification limits, assuming the process is centered on the target value. The higher the Cp, the better the process capability. In general, the interpretation of these indices is as follows:

- If *C*_*p*_< 1: the process is not capable of producing products within the specified tolerance, and significant improvements are needed.
- If 1 ≤ *C*_*p*_< 1.33: the process is marginally capable but may not meet customer requirements consistently.
- If 1.33 ≤ *C*_*p*_< 1.67: The process is reasonably capable, but further improvements can enhance consistency.
- If *C*_*p*_≥ 1.67: the process is considered capable, and it is likely to meet customer requirements consistently.

Furthermore, *C*_*pk*_ measures the capability of a process while accounting for how well the process is centered within the specification limits. It takes into consideration both process variation and centering. Interpreting the values of *C*_*pk*_, is likely the same as *C*_*p*_ with consideration of process centering. Therefore, any process with *C*_*pk*_ less than 1.67 centering is necessary for improvements.

The measurements of the 3D printed cylindrical part’s tolerance standard (tolerance, lower and higher limits) were selected according to common tolerance standards [40] and plastics molding component specifications [43]. A ±0.1 *mm* tolerance was chosen. The Multivariate PCI of FDM process was estimated based on this standard. A capability target index of 1 was used to evaluate the capability indices.

Moreover, the variation of each QC will be investigated alone in terms of its central tendency and dispersion parameters. By comparing the variation of QCs to each other, one can determine the size of each QC effect to the total variation associated during multivariate PCI estimation. Therefore, the mean and/or standard deviation of each QC will decrease/increase to see its effect on decreasing or increasing multivariate PCI. The percentage of increase or decrease will be set to 20 percent of the original values. Therefore, the percentage of deviation of MPCI of each combination from the original multivariate PCI will be reported. These percentages will be used to test the effect size of each QC data on the estimated multivariate PCI.

### 3.5 Capable tolerances

A capable tolerance and its limit deviations will be generated using a target capability index instead of calculating the process capability for a given tolerance. 1.67 was chosen as the desired capability index. Eq (6) was used to generate the process K index, which expresses how close the process is to the desired value and is a suitable indicator of process centering [44,45]. Eq (7) was used to estimate the capability tolerance’s lower (LSLT) and upper (USLT) specification limits. Then, using Eq (7), the upper limit deviation (ULD) and lower limit deviation (LLD) over the nominal dimension were calculated [6], where the constant K is calculated using Eq 6.

$$K=\frac{(USL+LSL)-2*X_{mean}}{USL-LSL}$$ (6)

Where *X*_*mean*_ is the median. If 0<|*K*|<1, the process mean is located between the middle of the specifications and one of the critical limits. If |*K*|>1, it means that the process mean is outside the necessary limits [6].

$$If\;K>0\;then\left\{ \begin{array}{c} {LSL}_{T}=X_{50\%}-C_{p}(X_{50\%}-X_{0.135\%})\\ LLD=T_{m}-{LLS}_{T}\\ {USL}_{T}=LSL+C_{p}(X_{99.865\%}-X_{0.135\%})\\ ULD={USL}_{T}-T_{m} \end{array}\right.$$ (7)

$$If\;K<0\;then\left\{ \begin{array}{c} {USL}_{T}=X_{50\%}+C_{p}(X_{99.865\%}-X_{50\%})\\ ULD={TUSL}_{T}-T_{m}\\ {LSL}_{T}=USL+C_{p}(X_{99.865\%}-X_{0.135\%})\\ LLD=T_{m}-{LSL}_{T} \end{array}\right.$$ (8)

where T_m_ is the tolerance center, X_0.135%_ is the 0.135% distribution quantile.

## 4. Research results

### 4.1 Printing process and data collection

The QCs of printed parts using the FDM process are height (12mm), diameter (14.5mm), and wall thickness (0.45mm). The specification limits for each QC were established according to ISO standard and according to the natural tolerances of the process. ISO 2768–1 standard [40] stated the fine, medium, coarse, and very coarse tolerances of linear dimensions for different size ranges. For example, a fine tolerance limit for dimensions between 6 mm and 30 mm is ±0.1 *mm*. Table 2 shows the nominal dimensions of the considered QCs and their upper and lower tolerances according to different designation of ISO tolerances, and natural tolerance of the process. The natural tolerances will be used as specification limits, in addition to the ISO standard, to evaluate the performance of the proposed method in estimating multivariate PCI. Therefore, the estimated multivariate PCI, according to natural tolerances, will be compared to the well-known PCI of three sigma process deviation. It is worth noting that the actual means of all QCs are not centered and always shifted toward the lower specification limits.

**Table 2:** Different specification limits designations.

QC	Nominal Dimensions	Specification limits according to ISO standard								Tolerance limits		Mean	STD
		Fine limits		Medium limits		Coarse limits		Very Coarse limits					
		USL	LSL	USL	LSL	USL	LSL	USL	LSL	USL	LSL		
Height (mm)	12	12.1	11.9	12.2	11.8	12.5	11.5	13	11	11.97	11.90	11.93	0.037
Diameter (mm)	14.5	14.6	14.4	14.7	14.3	15	14	15.5	13.5	14.48	14.34	14.41	0.074
Wall thickness (mm)	0.45	0.5	0.4	0.55	0.35	0.65	0.25	0.75	0.15	0.48	0.43	0.452	0.027

### 4.2 Statistical assumptions

**Fig 5:**
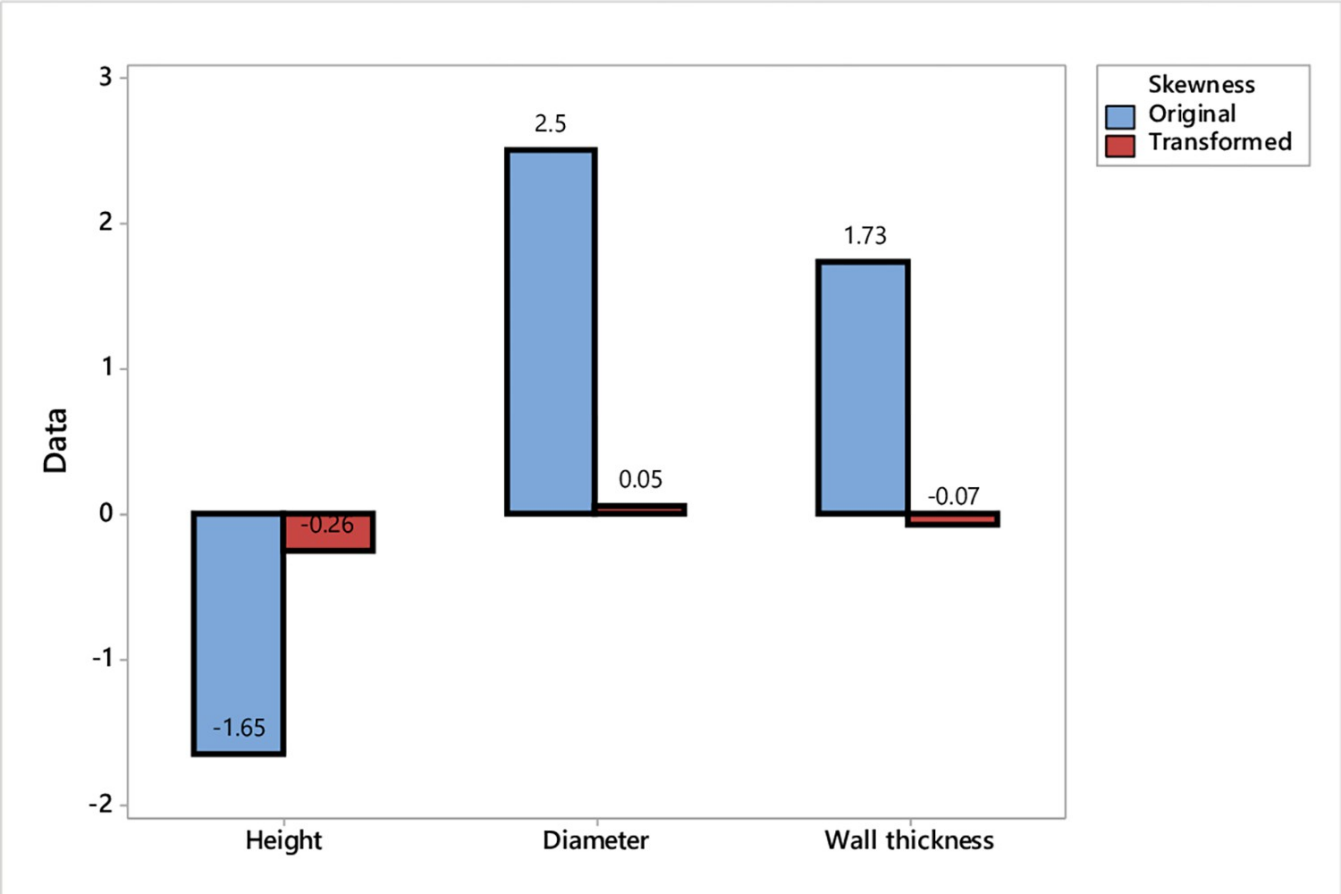
Original and transformed data skewness.

The normality assumption of collected data is tested for the three considered QCs: height, diameter, and wall thickness. Testing normality was conducted using skewness measures. The skewness results of height, diameter, and wall thickness are -1.65, 2.5, and 1.723, respectively. According to the rules mentioned in the methodology section, the data of the height, diameter and wall thickness are not normal. Therefore, the data of these QCs are transformed to normality using Johnson transformation method. As shown in Fig 5, the transformation method efficiently reduced the skewness of the considered data.

Since the original data of the QCs have been transformed to normality, the specifications limits will be transformed by the same method. Therefore, the utilized equations during the transformation process were used for specification limits transformation. The equations used to transform each QC data are as follows:

$$Ttransformed\;dta=\left\{ \begin{array}{c} 3.035+2.336*ASINH(\frac{X-11.989}{0.0308}),for\;Height\;data\\ -1.156+1.409*ASINH(\frac{X-14.3461}{0.0587}),for\;Diameter\;data\\ -0.876+0.88*ASINH(\frac{X-0.435}{0.0064}),for\;wall\;thickness\;data \end{array}\right.$$

The specification limits after transformation are presented in Table 3. The transformed specification limits will be used to estimate the MPCI of the process.

**Table 3:** Transformed specification limits.

QC	Nominal Dimensions	Specification limits according to ISO standard								Tolerance limits		Mean	STD
		Fine limits		Medium limits		Coarse limits		Very Coarse limits					
		USL	LSL	USL	LSL	USL	LSL	USL	LSL	USL	LSL		
Height (mm)	3.85	7.69	-1.1	9.16	-2.8	11.2	-5.05	12.81	-6.69	3.25	-3.10	0.08	1.06
Diameter (mm)	1.23	1.9	0	2.36	-2.2	3.22	-4.64	4.02	-5.89	2.78	-2.87	-0.04	0.94
Wall thickness (mm)	0.53	1.78	-3	2.28	-3.8	2.83	-4.45	3.17	-4.83	3.47	-3.32	0.07	1.13

### 4.3 Estimating MPCI

The normal data of QCs are standardized for the purpose of estimating the MPCI of the process. The standard variables have zero mean and one variance. Since three considered QCs are considered in this research, the mean of the data is the vector of 1 by 3 size (mean = [0 0 0]). The mean vector and covariance matrix are used to generate a large sample of multivariate normal distribution. The sample size of the simulated sample is 1,000,000 vectors of 3 variables. Each vector is tested against the specification vector, in which any data of the tested vector violates its corresponding specification limit; the whole vector is considered nonconforming. The simulation process is repeated 1000 times for each type of specification limit (fine, medium, coarse, and natural tolerances), then the average MPCI is estimated. Table 4 shows the MPCI for each specification type. To evaluate the performance of the proposed method, the MPCI with natural tolerances must be estimated mathematically. The nonconforming percent for the centered process and 1.5 *σ* using natural tolerances (3 sigma) are 0.0027 and 0.0668, respectively. Therefore, the actual PCI for a centered process and 1.5 *σ* shifted process are calculated as follows:

$${C_{p}}_{centered}=\frac{\varphi^{-1}(0.5+0.5(1-0.0027))}{3}=1$$

$${C_{p}}_{shifted}=\frac{\varphi^{-1}(0.5+0.5(1-0.0668))}{3}=0.611$$

**Table 4:** MPCIs for different specifications designations.

Specification type	Fine	Medium	Coarse	Very coarse	Natural tolerance
Estimated MPCI	0.16	0.67	0.89	0.99	0.88

The average shift of the considered process is 1.18, which is between the centered and 1.5 shifted processes. Therefore, the actual PCI of the process with 3 sigma tolerances is located between 0.611 and 1. As shown in Table 4, the estimated MPCI for natural tolerances is 0.88, which is between centered and 1.5 shifted processes. This results indicate that the proposed methodology for estimation MPCI is doing well. Moreover, Table 4 presents the results of MPCI for the considered process using different designations of specifications limits. Both fine and medium specifications are less than the natural tolerance, whereas the coarse and very coarse specification limits revealed an MPCI of 0.89 and 0.99, respectively, which are more than the MPCI estimated using natural tolerances. It is worth noting that all specification designations, except very coarse specification limits, showed that the considered process is not capable of producing products with the desired specifications. This is because all the estimated MPCIs are less than one. MPCI for very coarse limits is almost one, and the process is considered capable of producing parts conforming to very coarse specification limits.

## 5. Discussions

### 5.1 Capable tolerances

This section investigates the tolerances that the process is capable to meet. Capable tolerances are the tolerances that lead the process to have a capability index of more than one. Therefore, the process is evaluated according to the precisions of the capable tolerances. Capable tolerances were established according to Eqs 7 and 8. Table 5 shows the capable tolerances for each QC. Since the process has more than QC and estimates an MPCI, the capable tolerance is unique for all QCs. Such that the lower limit deviation (LLD) and upper limit deviation (ULD) for each QC are calculated, then the minimum LLD of all QCs is subtracted from the maximum ULD of all QCs. This results in a single tolerance for all QCs is called capable tolerance (Tc). Table 5 shows that the capable tolerance of producing one capability index is 0.64. Using capable tolerance, lower capable specification limits (LSLc) and upper capable specification limits (USLc) of each QC are calculated, which is the nominal dimension ± capable tolerance. Using the capable specification limits, MPCI is estimated in the same way, by simulating large samples from the normal distribution using QCs data properties. The estimated MPCI is 1.15, as shown in Table 5; however, it is supposed to be one. This small deviation is due to the associated randomness during data generation.

**Table 5:** Capable tolerance and capable specifications.

QC	K	LLDi	ULDi	LSLc	USLc	Tc	Cp
Height-	0.12	0.20	0.37	11.36	12.64	0.64	1.15
Diameter-	0.21	0.20	0.67	13.86	15.14		
Wall thickness-	0.03	0.03	0.14	-0.19	1.09		

### 5.2 Analyzing of variations

This section aims to quantify the process variation for different process QCs. Therefore, the coefficient of variation (CV) measure was used, as presented in Table 6. The CV values revealed that the most variation was associated with wall thickness characteristics data. The percentage of wall thickness variation is about 6%, whereas CVs for height and diameter are less than 1% for both. Therefore, the appropriate corrective action is to investigate the process variables that may affect wall thickness accuracy. Moreover, shifts in the process exist for each QC. Such that height and diameter QCs have means less than their nominal values, whereas wall thickness data revealed a mean slightly thicker than the nominal wall thickness. To evaluate the amounts of shifts in the process for each QC, the collected data of each QC were compared to its nominal dimension using one sample t-test, as shown in Table 6. The results of the t-test would be interpreted using p-values at a significant level of a = 0.05. P-values less than a significant level indicate a significant difference between the sample data and the hypothesized mean. Therefore, the height data are significantly different from the nominal height (12 mm). Also, the diameter data significantly differs from the nominal diameter (14.5 mm). At the same time, the wall thickness data are not significantly different from the nominal wall thickness (0.45 mm), which revealed that the process has shifted from the height and diameter targets. Consequently, the investigation should focus on adjusting the mean height and diameter QCs. Although the dispersion in wall thickness data is greater than the dispersion in other QCs (height and diameter), wall thickness data are not shifted from the nominal value of height and diameter data. This is because the wall thickness is more scattered around the nominal wall thickness value, whereas the height and diameter data are less scattered but away from their nominal values.

**Table 6:** Variations and shifts from nominal dimensions.

QC	Nominal	Mean	CV	95% CI	T	P-value
Height (mm)	12	11.933	0.31%	(11.9226, 11.9434)	-12.91	0
Diameter (mm)	14.5	14.41	0.51%	(14.3890, 14.4310)	-8.61	0
Wall thickness (mm)	0.45	0.452	5.95%	(0.44436, 0.45964)	0.53	0.601

### 5.3 Sensitivity analysis

The sensitivity analysis has been investigated to present the effect of the variation and mean shift of each QC. The sensitivity analysis was established by increasing/decreasing the mean and/or standard deviation of each QC. The percent of increase or decrease of mean or standard deviation is 50 percent. A total of 24 runs was simulated by increasing/ decreasing the mean or standard deviation of each of the three QCs (12 runs), both the mean and standard deviation of each QC (6 runs), the mean of all QCs together (2 runs), the standard deviation of all QCs (2 runs), and both the mean and the standard deviation of all QCs (2 runs).

**Fig 6:**
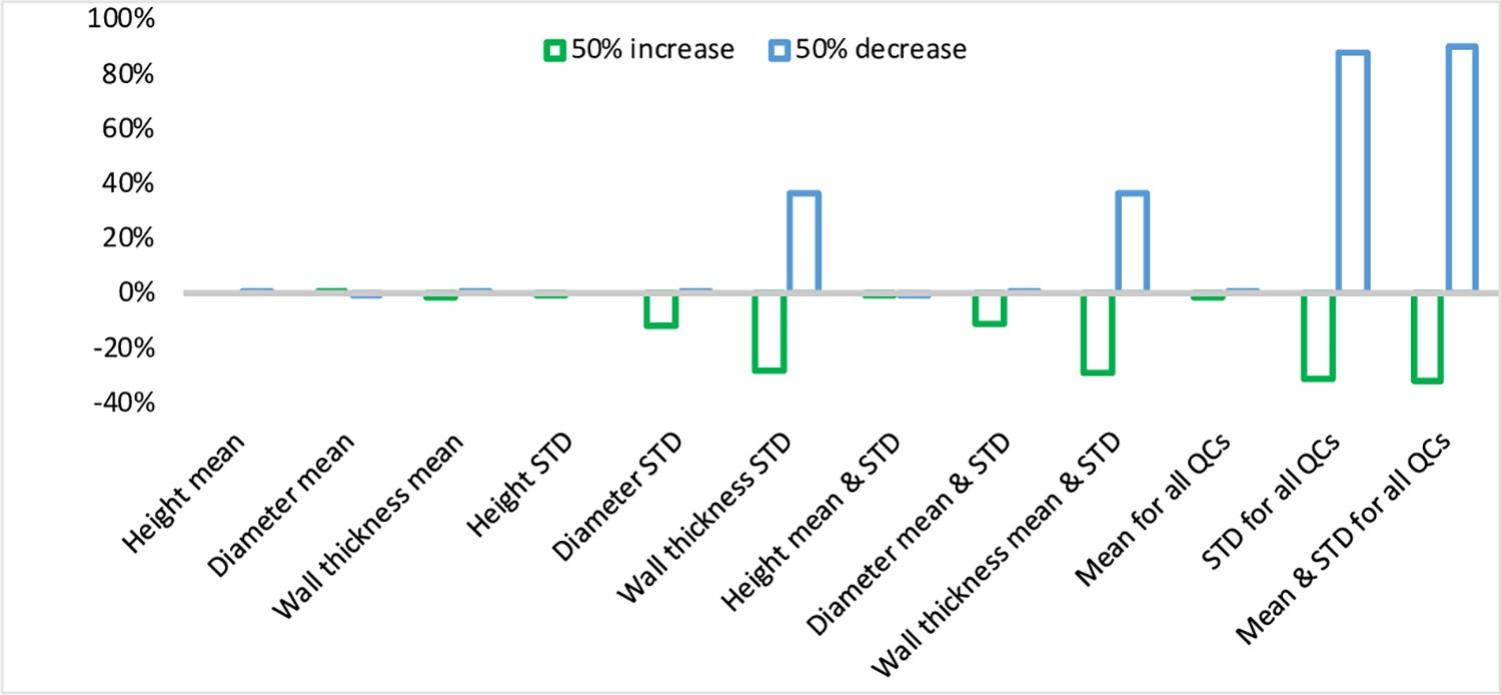
Sensitivity analysis.

Fig 6 shows the results of the changes in MPCI while increasing or decreasing mean or standard deviation or both mean and standard deviation for each or all QCs. Each time change/s to mean and/or standard deviation are done, MPCI is estimated according to coarse specification limits, and the percent change in the MPCI is calculated as follows:

$$\frac{New\;MPCI-0.89}{0.89}*100\%$$

Therefore, the MPCIs greater than 0.89 will result in a positive change percent, whereas a lower MPCI produces a negative change percent. The results in Fig 6 show that changing the mean for all QCs has a slight or no effect on estimating MPCI. Consequently, a single change of mean is not sensitivewhen estimating MPCIs, as shown by the first bars of the graph. The apparent changes in MPCI started by changing the standard deviation. Increasing the standard deviation for each QC continuously decreases MPCIs, while decreasing the standard deviation increases MPCIs. The most change for a single change of standard deviation is noticed while decreasing standard deviation for wall thickness data, resulting in an increase in MPCI by 37%. Therefore, the standard deviation is sensitive to changes while estimating MPCI. However, changing both mean and standard deviation has no additional effect. Also, increasing or decreasing the mean for all QCs has a negligible effect on MPCI estimation. Moreover, changing the standard deviation for all QCs greatly effects MPCI. Decreasing the standard deviation for all QCs increases estimated MPCI by about 90%, while increasing standard deviation for all QCs decreases MPCI by only 31%. Again, the effect of increasing/decreasing the mean for all QCs is negligible.

### 5.4 Managerial insights

The application of MPCA is particularly relevant in the evaluation of AM processes, where multiple quality characteristics often need to be assessed simultaneously. In additive manufacturing, various factors such as material properties, geometric features, surface finish, and dimensional accuracy contribute to the overall quality of the manufactured components. Evaluating these multivariate quality characteristics collectively provides a more comprehensive understanding of process performance and product quality. By employing MPCI, additive manufacturing practitioners can assess the capability of their processes to meet the desired specifications across multiple dimensions. This involves collecting and analyzing data on these quality characteristics to estimate joint distribution and variation parameters. Through MPCI, stakeholders can gain insights into how well the AM process performs in producing components that meet the required quality standards across all relevant dimensions.

However, implementing MPCI in the context of AM comes with its own set of challenges, such as ensuring the collection of sufficient and representative data from the manufacturing process. Additionally, effectively communicating and interpreting the results of MPCA to stakeholders, including designers, engineers, and decision-makers, is crucial for informed decision-making and process improvement efforts. Overall, the integration of MPCA into the evaluation process of AM processes enables a comprehensive assessment of QCs, leading to improved process control, enhanced product quality, and ultimately, increased customer satisfaction.

## 6. Conclusion

A framework for evaluating AM processes using MPCI has been presented in this research. The research is based on the FDM process as a leading AM process that could be an excellent example for evaluating AM processes. In the evaluation process, dimensional accuracy is a proper critical QC that can be used to judge the capability of AM processes. To measure AM process capability of producing the desired specification, process capability indices are recommended since they are widely used and have been proven for their estimation efficiency. The results showed that the FDM process is not capable of producing parts with fine, medium, and coarse limits, while it can produce parts with very coarse limits. However, the process could be investigated under coarse specification limits followed by process improvement. The process data were tested for normality assumption, and the results showed that the data were not normally distributed. Therefore, transforming non-normal data is crucial before continuing with the estimation of the process capability index. By ensuring the normality of the data, estimating MPCI using PNC is a practical approach. However, it needs a very large sample size and a large number of iterations, which are conducted using Matlab software. The values of MPCI revealed in this research showed that the considered FDM process is not capable of producing parts within suggested designation of specification limits. To show the gap between the suggested limits and the limits that the process is capable to meet, a capable tolerance has been calculated. Since the process capability is estimated using multivariate QCs, the effect of each QC is analyzed using mean shift and standard deviation. This showed that among three used QCs, two QCs (height and diameter) means are shifted and statistically different from the nominal values. Consequently, the sensitivity analysis for mean and standard deviation is conducted, which showed that changing standard deviation is more sensitive than changing means. The research conducted in this study is based on constant process parameters of FDM. In this regard, a promising research direction is to optimize the process parameters based on the values of MPCI.

## Supporting information

S1 Data Original and processed excel file. (XLSX)
